# Academic procrastination and academic engagement among dentistry students at a Peruvian university: a cross-sectional study

**DOI:** 10.3389/fpsyg.2026.1881748

**Published:** 2026-09-08

**Authors:** Dorothy Luisa Meléndez Morote, Alex David Vidal Mosquera, Heiden Peche Buquez

**Affiliations:** Programa de Odontología, Universidad de San Martín de Porres, Chiclayo, Peru

**Keywords:** academic engagement, academic procrastination, dentistry, engagement, self-regulation, university students

## Abstract

**Introduction:**

Academic procrastination is a common self-regulatory difficulty in higher education and may be related to how strongly students engage with academic work. This study examined the association between academic procrastination and academic engagement among dental students at a Peruvian university. Methods: We conducted a cross-sectional analytical study using 426 complete records from the Lima, Chiclayo, and Arequipa campuses of Universidad de San Martín de Porres. Academic procrastination was represented by a 12-item PASS-derived score, and academic engagement by the 17-item UWES-Student total score and its vigor, dedication, and absorption dimensions. Internal consistency was estimated with Cronbach’s alpha, and associations were evaluated using Spearman’s rho with 95% bootstrap confidence intervals.

**Results:**

Cronbach’s alpha was 0.954 for the procrastination score, 0.962 for total engagement, 0.912 for vigor, 0.883 for dedication, and 0.912 for absorption. Higher procrastination was moderately associated with lower total engagement (rho = −0.424, 95% CI -0.503 to −0.341; *p* < 0.001) and lower vigor (rho = −0.425, 95% CI -0.503 to −0.341; *p* < 0.001). Inverse associations were also observed for dedication (rho = −0.397, 95% CI -0.475 to −0.309; *p* < 0.001) and absorption (rho = −0.385, 95% CI -0.464 to −0.298; *p* < 0.001).

**Conclusion:**

Greater academic procrastination was consistently associated with lower academic engagement in this multicampus sample. Because the study was cross-sectional and based on self-report, the findings do not establish causality or demonstrate that any form of procrastination is adaptive.

## 1 Introduction

Academic procrastination, defined as the unnecessary and voluntary delay of academic tasks despite expecting negative consequences, is a frequent problem in higher education and has been associated with lower academic performance, reduced self-regulation, and psychological discomfort among university students from different disciplines (Wei et al., 2025; Araya-Castillo et al., 2023). From the perspective of Temporal Motivation Theory, procrastination emerges when students prioritize the immediate reward of postponing a task over the delayed benefits of completing it, especially when task value, expectancy of success, and self-regulatory capacity are low (Adeel et al., 2023; Siaputra, 2010). This is mainly observed in students who lack self-efficacy and fear failure, which increases procrastination (Huanambal-Pérez et al., 2026; López et al., 2024). This behavior is particularly evident in students with low academic self-efficacy, fear of failure, poor time management, or difficulty regulating motivation, all of which may increase the tendency to postpone academic responsibilities.

Academic engagement represents a central construct for understanding students’ involvement in their learning process. It is commonly understood as a positive academic state characterized by vigor, dedication, and absorption. Vigor refers to energy and persistence during academic activities; dedication involves enthusiasm, meaning, and commitment to studies; and absorption refers to concentration and immersion in academic tasks. In this sense, procrastination and engagement may be theoretically opposed constructs, since delaying academic activities can interfere with students’ behavioral, emotional, and cognitive involvement in learning (Wang and Wang, 2024). However, this relationship may not always be linear, as some students may maintain moderate or high engagement despite occasionally postponing tasks.

In demanding academic programs such as dentistry, the study of procrastination and engagement is especially relevant. Dental education requires students to integrate theoretical knowledge, laboratory training, clinical procedures, ethical responsibility, and patient care, creating a highly demanding academic environment (Song et al., 2024; Sirois, 2023). These conditions may increase stress, academic overload, and difficulty managing time, which can contribute to procrastination. At the same time, the professional and clinical nature of dental training may also promote engagement, since students are progressively exposed to practical responsibilities that demand concentration, persistence, and commitment. Therefore, dental students may present complex profiles in which procrastination coexists with different levels of academic engagement.

Previous studies have reported that procrastination is common among dental and health sciences students, with estimates ranging from moderate to high prevalence depending on the population and measurement approach (Uma et al., 2020; Rozental et al., 2022). The causes of this phenomenon are multifactorial and include psychological factors, such as anxiety, fear of failure, and low self-efficacy; academic factors, such as workload and evaluation pressure; and contextual factors, such as clinical demands and institutional expectations. In dental students, persistent procrastination may not only affect academic performance but also interfere with the development of professional competencies, particularly when delayed preparation limits participation in theoretical, preclinical, or clinical activities (Babayiğit et al., 2024).

Self-efficacy, motivation, and academic engagement have been identified as factors related to procrastination. Students who perceive greater competence, meaning, and motivation in their academic activities may be less likely to postpone tasks in a maladaptive manner (Ma and Chen, 2024; González-Brignardello et al., 2023). Rather than claiming a systematic absence of evidence across Latin America, the present study addresses a narrower, context-specific question: whether a PASS-based procrastination score is associated with overall academic engagement and with vigor, dedication, and absorption among dental students enrolled across three campuses of a Peruvian university.

Understanding this association can inform student-support strategies focused on self-regulation, time management, motivation, and academic persistence (Gayary and Kalita, 2025; Mori and García, 2022). The study therefore aimed to determine the association between academic procrastination and academic engagement among dental students at Universidad de San Martín de Porres in Lima, Chiclayo, and Arequipa during 2025. Secondary objectives were to evaluate the associations between procrastination and the engagement dimensions of vigor, dedication, and absorption. We hypothesized that higher procrastination scores would be associated with lower engagement scores.

## 2 Materials and methods

### 2.1 Study design and setting

A quantitative, non-experimental, cross-sectional analytical study was conducted among students enrolled in the Dentistry Program at Universidad de San Martín de Porres during the 2025 academic period. Data were collected at the Lima, Chiclayo, and Arequipa campuses. Academic procrastination was the explanatory construct, while academic engagement was evaluated as the overall UWES-Student score and through vigor, dedication, and absorption. Because exposure and outcome were measured at the same time, the analyses were designed to estimate associations rather than causal effects.

### 2.2 Source population, sample size, and analytical sample

The source population consisted of 1,400 students enrolled from the first through tenth academic cycles across the three campuses. Eligible participants were officially enrolled during the 2025 academic period, belonged to one of the participating campuses, and voluntarily provided informed consent. Questionnaires with incomplete item responses, duplicated records, inconsistent response patterns, or refusal to participate were not eligible for the analytical dataset.

The initial project had a target sample size of 624 participants, with a 95% confidence level and an absolute precision of 5%; however, there were limitations in accessing the target population and meeting the inclusion criteria. For transparent reproducibility, the conventional formula for finite populations was used: *n* = [*N* × *Z*^2^ × *p*(1-p)]/[*d*^2^ × (N-1) + Z^2^ × p(1-p)], using *N* = 1,400, *Z* = 1.96, *p* = 0.50, and d = 0.05, yields 301.6 participants, rounded to 302. The final analytical dataset contained 426 complete and unique records: 95 from Lima, 173 from Chiclayo, and 158 from Arequipa.

The study selected participants using non-probability census sampling based on the inclusion criteria. The 426 records analyzed represent 30.4% of the original population, which consisted of 1,400 students.

### 2.3 Variables and instruments

Academic procrastination was assessed using data recorded under the Academic Procrastination Assessment Scale (PASS), originally developed by Solomon and Rothblum (1984). The study database contains 43 PASS-related items coded from 1 to 5. The first 18 items comprise three ratings for each of six academic activities. The primary procrastination score used in the present analysis sums the first two ratings for each activity (12 items; possible range 12–60), with higher values indicating greater procrastination. The six desire-to-change ratings were retained separately, and the remaining 25 items concerning reasons for delay were not included in the primary procrastination score (Vitória et al., 2018).

Academic engagement was assessed using the 17-item UWES-Student form. In the analytical dataset, all 17 items were coded from 1 to 5, producing a total score ranging from 17 to 85. Vigor was calculated from six items (possible range 6–30), dedication from five items (5–25), and absorption from six items (6–30); higher scores indicate greater engagement. Internal consistency was estimated using Cronbach’s alpha for the 12-item procrastination score, the 17-item engagement score, and the three engagement dimensions. In the analysis, these variables were treated as continuous composite scores.

### 2.4 Data collection procedure

Data were collected through structured, self-administered questionnaires applied in person at the Lima, Chiclayo, and Arequipa campuses during the 2025 academic period. Before completing the questionnaires, students received information about the objective of the study, the voluntary nature of participation, the confidentiality of their responses, and their right to withdraw at any time without academic consequences.

The questionnaires were administered under standardized conditions to reduce information bias and ensure that all participants received the same instructions. Students completed the instruments individually and without time pressure. The research team reviewed the questionnaires after collection to identify incomplete or invalid responses. Only questionnaires with complete and consistent information were included in the final database.

The collected information was coded and entered into a database for statistical analysis. Participants’ identities were not recorded in the analytical database, and each questionnaire was assigned a numerical code to preserve anonymity.

### 2.5 Statistical analysis

Data were analyzed using IBM SPSS Statistics version 26.0 and Python 3.13 (NumPy 2.3.5 and SciPy 1.17.0). The set of analytical data was independently verified to check its integrity, the presence of duplicate identifiers, the ranges of the questions, and the reconstruction of the scores. Continuous composite scores are summarized using mean and standard deviation together with median, interquartile range, and observed range. Cronbach’s alpha was calculated from the items composing each primary score. The primary inferential analysis used Spearman’s rank correlation coefficient (rho) to evaluate the association of academic procrastination with overall engagement, vigor, dedication, and absorption. This approach was selected because it estimates monotonic association without requiring normally distributed scores and is appropriate for bounded composite scores derived from ordinal responses. Two-sided *p* values below 0.05 were considered statistically significant. Ninety-five percent confidence intervals for rho were obtained using percentile bootstrap resampling with 10,000 replicates. Campus-stratified Spearman correlations were calculated as exploratory sensitivity analyses and are reported in the Supplementary material.

### 2.6 Ethical considerations

This study was conducted in accordance with the ethical principles for research involving human participants and followed the guidelines of the Declaration of Helsinki. Participation was voluntary, and all students provided informed consent before answering the questionnaires. Participants were informed about the purpose of the study, the procedures involved, the confidential handling of the data, and their right to withdraw from the study at any time without consequences.

Confidentiality and anonymity were guaranteed throughout the research process. No identifying information was included in the final database, and the data were used exclusively for academic and scientific purposes. The study was approved by the Ethics Committee of Universidad de San Martín de Porres, under approval code No. 013-2023-VRII-USMP.

## 3 Results

### 3.1 Descriptive analysis

The final analytical sample comprised 426 complete and unique records: 95 students from Lima (22.3%), 173 from Chiclayo (40.6%), and 158 from Arequipa (37.1%). No missing PASS or UWES item values were present in the analytical dataset. The median procrastination score was 34 (IQR 25–42), and the median overall engagement score was 56.5 (IQR 43.25–67). Internal consistency was high for the procrastination and engagement measures, with alpha values ranging from 0.883 to 0.962 (Table 1).

**Table 1:** Descriptive statistics and internal consistency of the study scores.

Measure	Mean	SD	Median	IQR	Observed range	Cronbach alpha
Academic procrastination	34.25	11.38	34.0	25.0–42.0	12–60	0.954
Academic engagement	55.50	15.66	56.5	43.25–67.0	17–85	0.962
Vigor	19.58	5.83	20.0	16.0–24.0	6–30	0.912
Dedication	16.20	4.77	16.0	13.0–20.0	5–25	0.883
Absorption	19.72	5.88	20.0	16.0–24.0	6–30	0.912

Academic procrastination showed a statistically significant inverse association with overall academic engagement (rho = −0.424, 95% CI -0.503 to −0.341; *p* < 0.001). Similar inverse associations were observed for vigor (rho = −0.425, 95% CI -0.503 to −0.341; *p* < 0.001), dedication (rho = −0.397, 95% CI −0.475 to −0.309; *p* < 0.001), and absorption (rho = −0.385, 95% CI −0.464 to −0.298; *p* < 0.001) (Table 2). The direction of association was also negative in exploratory analyses within each campus (Supplementary Table S3).

**Table 2:** Spearman correlations between academic procrastination and academic engagement.

Outcome	Spearman rho	95% CI lower	95% CI upper	*p* value
Academic engagement	−0.424	−0.503	−0.341	<0.001
Vigor	−0.425	−0.503	−0.341	<0.001
Dedication	−0.397	−0.475	−0.309	<0.001
Absorption	−0.385	−0.464	−0.298	<0.001

Confidence intervals are percentile bootstrap intervals based on 10,000 resamples. All tests were two-sided.

## 4 Discussion

In this multicampus sample of dental students, higher academic procrastination was consistently associated with lower academic engagement. The association was moderate for total engagement and vigor and somewhat smaller for dedication and absorption. These results directly address the primary and secondary study objectives and replace the earlier interpretation based mainly on percentages and undocumented categorical cutoffs. The findings support an inverse monotonic relationship between the two constructs, while the cross-sectional design limits inference to association.

The direction of the observed associations is consistent with literature linking procrastination to weaker learning involvement, lower self-regulation, academic stress, and less favorable educational outcomes in university and health-sciences populations (Huanambal-Pérez et al., 2026; Wang and Wang, 2024; Uma et al., 2020; Babayiğit et al., 2024; Yang et al., 2023). At the same time, the magnitude of the correlations indicates that procrastination and engagement are related but not interchangeable constructs. Students with similar procrastination scores may still differ in motivation, self-efficacy, workload, clinical responsibilities, and coping resources, which may partly explain the residual variability in engagement.

The concept of adaptive or active procrastination warrants careful interpretation. Active procrastination generally refers to intentional delay accompanied by perceived control and preserved performance under time pressure, whereas passive procrastination reflects unwanted delay and self-regulatory difficulty. A recent meta-analysis distinguishes these patterns and cautions against treating all delay as functionally equivalent (Kooren et al., 2024). Ahmed et al. (2023) likewise emphasize the prevalence and negative academic consequences of procrastination in students. Importantly, the present study did not measure intention to delay, perceived control over deadlines, time–pressure preference, or performance consequences; therefore, coexistence of procrastination and engagement in some students cannot be interpreted as evidence of adaptive procrastination. Interventions should avoid normalizing delay solely because a student remains engaged and should instead consider the degree of control, distress, and functional impairment associated with the behavior.

For educators and academic administrators, the results support a risk-based rather than purely categorical approach. Programs may consider early identification of students who simultaneously report high procrastination and low engagement, structured interim deadlines for complex assignments, explicit training in planning and self-regulation, mentoring during transitions to preclinical and clinical coursework, and coordination of assessment schedules to reduce avoidable workload clustering. These strategies should be implemented as supportive educational measures rather than punitive responses. Because procrastination has been associated with subsequent health outcomes, academic pressure, and self-efficacy-related well-being in university populations (Johansson et al., 2023; Xue et al., 2025; Demir and Kuşcu Karatepe, 2025), student-support programs should also provide pathways to counseling when procrastination co-occurs with substantial stress or emotional distress. In dental education, attention to students’ motives, time management, resilience, and engagement may be particularly relevant because academic responsibilities progressively interact with clinical obligations and patient care (Shin et al., 2024; Ahmad et al., 2024; Zhang, 2021).

Several limitations should be considered. First, the cross-sectional design precludes temporal or causal inference. Second, all variables were self-reported, which may introduce common-method and social-desirability bias; students may underreport procrastination or over report engagement. Anonymous coding, standardized self-administration, and the absence of identifying information may have reduced evaluation pressure, but they cannot eliminate this bias. Third, participants came from a single university, albeit from three campuses, so the findings should not be generalized automatically to other dental schools or countries. Fourth, the census-based participant selection approach precluded verification and calculation of the response rate. Fifth, the study did not employ specific locally adapted versions of the PASS and UWES-Student instruments. Finally, demographic characteristics, academic cycle, academic performance, and psychosocial covariates were not prioritized in the study design, which prevented adjusted analyses for potential confounding factors or effect modification.

The study nevertheless has several strengths: it includes 426 students across three campuses, the primary scores showed high internal consistency, and the revised analysis uses continuous scores with correlation coefficients, confidence intervals, and exact directionality rather than relying on descriptive cross-tabulations alone. Future studies should use prospectively documented probability-based or clearly described recruitment procedures, validated Spanish-language instrument versions, and repeated measurements across academic cycles. Longitudinal studies could clarify temporal ordering, while randomized or quasi-experimental evaluations of self-regulation, planning, or time-management interventions could test whether reductions in procrastination are followed by sustained improvements in engagement. Multi-institutional studies should also evaluate whether the association differs by clinical stage, workload, sex, age, academic performance, or mental-health variables.

## 5 Conclusion

Among 426 dental students from three campuses of a Peruvian university, greater academic procrastination was associated with lower overall academic engagement and with lower vigor, dedication, and absorption. The associations were statistically significant, with moderate coefficients for total engagement and vigor and somewhat smaller coefficients for dedication and absorption. These findings support the relevance of self-regulation and engagement-oriented student support, but they do not establish that procrastination causes disengagement. The study also did not measure active procrastination and therefore cannot determine whether any observed delay was adaptive. Longitudinal and intervention-based research is needed to evaluate temporality and the effects of targeted self-regulation strategies over time.

## Data Availability

The original contributions presented in the study are included in the article/Supplementary material, further inquiries can be directed to the corresponding author.
