# Supplementary material for: Academic procrastination and academic engagement among dentistry students at a Peruvian university: a cross-sectional study
Source: Front Psychol. 2026 Sep 8;17:1881748. doi: 10.3389/fpsyg.2026.1881748 (PMC13593870; doi:10.3389/fpsyg.2026.1881748)
Supplement: Supplementary file 1 [file Table_2.DOCX]

Supplementary Material

**Table S1. Scoring specification for the analytical variables.**

| **Construct** | **Items used** | **Coding** | **Possible range** | **Direction** | **Role in analysis** |
| --- | --- | --- | --- | --- | --- |
| Academic procrastination | PASS_01, 02, 04, 05, 07, 08, 10, 11, 13, 14, 16, 17 | 1-5 | 12-60 | Higher = greater procrastination | Primary explanatory score |
| Desire to change | PASS_03, 06, 09, 12, 15, 18 | 1-5 | 6-30 | Higher = greater desire to change | Not used in primary association analysis |
| Reasons for delay | PASS_19-PASS_43 | 1-5 | 25-125 | Higher = greater summed endorsement | Not used in primary association analysis |
| Academic engagement | UWES_01-UWES_17 | 1-5 | 17-85 | Higher = greater engagement | Primary outcome score |
| Vigor | UWES_01, 04, 08, 12, 15, 17 | 1-5 | 6-30 | Higher = greater vigor | Secondary outcome |
| Dedication | UWES_02, 05, 07, 10, 13 | 1-5 | 5-25 | Higher = greater dedication | Secondary outcome |
| Absorption | UWES_03, 06, 09, 11, 14, 16 | 1-5 | 6-30 | Higher = greater absorption | Secondary outcome |

*Note: The analytical file contains 43 PASS-related items and 17 UWES-Student items.*

**Table S2. Descriptive statistics, internal consistency, and pooled associations (n=426).**

| **Measure** | **Mean** | **SD** | **Median** | **IQR** | **Range** | **Cronbach alpha** | **Spearman rho vs PASS** | **p value** |
| --- | --- | --- | --- | --- | --- | --- | --- | --- |
| Academic procrastination | 34.25 | 11.38 | 34.0 | 25.0-42.0 | 12-60 | 0.954 | — | — |
| Academic engagement | 55.50 | 15.66 | 56.5 | 43.25-67.0 | 17-85 | 0.962 | -0.424 | <0.001 |
| Vigor | 19.58 | 5.83 | 20.0 | 16.0-24.0 | 6-30 | 0.912 | -0.425 | <0.001 |
| Dedication | 16.20 | 4.77 | 16.0 | 13.0-20.0 | 5-25 | 0.883 | -0.397 | <0.001 |
| Absorption | 19.72 | 5.88 | 20.0 | 16.0-24.0 | 6-30 | 0.912 | -0.385 | <0.001 |

*Note: Spearman correlations compare the 12-item academic procrastination score with each engagement outcome. Corresponding 95% percentile bootstrap confidence intervals were: engagement, -0.503 to -0.341; vigor, -0.503 to -0.341; dedication, -0.475 to -0.309; absorption, -0.464 to -0.298. Confidence intervals used 10,000 bootstrap resamples.*

**Table S3. Exploratory campus-stratified Spearman correlations.**

| **Campus** | **n** | **Outcome** | **Spearman rho** | **p value** |
| --- | --- | --- | --- | --- |
| Lima | 95 | Academic engagement | -0.357 | <0.001 |
| Lima | 95 | Vigor | -0.360 | <0.001 |
| Lima | 95 | Dedication | -0.313 | 0.002 |
| Lima | 95 | Absorption | -0.319 | 0.002 |
| Chiclayo | 173 | Academic engagement | -0.461 | <0.001 |
| Chiclayo | 173 | Vigor | -0.478 | <0.001 |
| Chiclayo | 173 | Dedication | -0.436 | <0.001 |
| Chiclayo | 173 | Absorption | -0.415 | <0.001 |
| Arequipa | 158 | Academic engagement | -0.406 | <0.001 |
| Arequipa | 158 | Vigor | -0.391 | <0.001 |
| Arequipa | 158 | Dedication | -0.396 | <0.001 |
| Arequipa | 158 | Absorption | -0.392 | <0.001 |

*Note: Campus-stratified analyses are exploratory sensitivity analyses. They do not establish representativeness within campuses and should not be interpreted as adjusted effects.*

**Data-integrity checks**

• All 426 identifiers were unique.

• No missing PASS or UWES item values were present.

• All PASS and UWES item responses were within the coded range 1-5.
